# Discovery and characterization of two Nimrod superfamily members in *Anopheles gambiae*

**DOI:** 10.1179/204777213X13867543472674

**Published:** 2013-12

**Authors:** Janet Midega, Joshua Blight, Fabrizio Lombardo, Michael Povelones, Fotis Kafatos, George K Christophides

**Affiliations:** 1 Department of Life Sciences, Imperial College London, UK; 2 Pathogen, Vector and Human Biology Unit, KEMRI–University of Oxford–Wellcome Trust Research Programme, Kilifi, Kenya; 3 The Jenner Institute, Nuffield Department of Clinical Medicine, University of Oxford, UK; 4 Division of Parasitology, Department of Public Health and Infectious Diseases, La Sapienza University of Rome, Italy; 5 Department of Pathobiology, School of Veterinary Medicine, University of Pennsylvania, Philadelphia, PA, USA

**Keywords:** *Anopheles gambiae*, Innate immunity, Microbiota, Phagocytosis, Nimrod superfamily, NIM repeats, EGF-like repeats

## Abstract

Anti-bacterial proteins in mosquitoes are known to play an important modulatory role on immune responses to infections with human pathogens including malaria parasites. In this study we characterized two members of the *Anopheles gambiae* Nimrod superfamily, namely AgNimB2 and AgEater. We confirm that current annotation of the *An*. *gambiae* genome incorrectly identifies AgNimB2 and AgEater as a single gene, AGAP009762. Through *in silico* and experimental approaches, it has been shown that AgNimB2 is a secreted protein that mediates phagocytosis of *Staphylococcus aureus* but not of *Escherichia coli* bacteria. We also reveal that this function does not involve a direct interaction of AgNimB2 with *S. aureus*. Therefore, AgNimB2 may act downstream of complement-like pathway activation, first requiring bacterial opsonization. In addition, it has been shown that AgNimB2 has an anti-*Plasmodium* effect. Conversely, AgEater is a membrane-bound protein that either functions redundantly or is dispensable for phagocytosis of *E. coli* or *S. aureus*. Our study provides insights into the role of members of the complex Nimrod superfamily in *An. gambiae*, the most important African vector of human malaria.

## Introduction

In comparison to vertebrates, invertebrates lack an adaptive immune system and rely solely on their innate immune response for defending against pathogens including the malaria parasite *Plasmodium falciparum*,^1^ bacteria, fungi, and viruses.^2^ *Anopheles gambiae* mosquitoes, the major vectors of *P. falciparum* malaria in sub-Saharan Africa, become infected with the malaria parasite following blood feeding on an infected human host and transmit the parasite during subsequent blood-meals.^3^,^4^ Within the vector, malaria parasites undergo various developmental transitions, the most critical being when ookinetes transform into oocysts.^1^,^5^ During this phase, parasites encounter many effectors of the mosquito innate immune system, including reactions such as melanization^6^ and lysis,^7^ which can lead to a massive reduction in parasite numbers or total blockade of transmission. The effectiveness of the immune responses targeting ookinetes is a major determinant of *Anopheles* vectorial capacity.^1^,^5^ Transmission only occurs if the parasites can evade these reactions and develop within the oocyst into sporozoites, the stage that is transmissible to humans.

The *An. gambiae* immune response to bacteria and other microbes consists of cellular and humoral arms. Following detection via pathogen recognition receptors (PRRs), the immune system responds with the cellular arm, through microbial phagocytosis, melanization, or lysis.^1^,^2^,^8^ Additionally, the humoral arm, also initiated by PRRs, produces soluble mediators such as anti-microbial peptides (AMPs) via the REL1- and REL2-signaling pathways.^1^,^2^,^9^ A major role in modulating anti-parasitic innate immune responses is played by gut microbiota, which induce immune effectors that also affect *Plasmodium,* for instance AMPs.^5^,^10^ The number of bacteria inducing anti-*Plasmodium* immune response rises dramatically following the intake of blood-meals by mosquitoes, thus ‘priming’ the immune response against the parasite.^5^

Phagocytosis is one of the most rapidly induced responses to microbial infection in mosquitoes, and therefore plays an important role in host defense.^2^ Phagocytic receptors may bind pathogens via the recognition of microbe associated molecular patterns (MAMPs). In addition, some phagocytic receptors depend on pathogen opsonization to bind and mediate phagocytosis.^2^,^8^,^11^ In either case, pathogens decoration by phagocytic receptors ultimately results in engulfment and killing by hemocytes. Understanding this important process is essential for further elucidation of the crosstalk between immune responses to bacteria and *Plasmodium*. A major obstacle in achieving this goal, is the paucity of information on phagocytic receptors and their pathways in *An. gambiae*.^2^,^8^,^12^ In this study, we characterized two members of the Nimrod superfamily in *An. gambiae* as members of this protein family have been shown to play an important role in bacterial phagocytosis in *Drosophila melanogaster.*^13^,^14^

## Materials and Methods

### AGAP009762 bioinformatic analysis

*An. gambiae* and *D. melanogaster* genome sequences were obtained from VectorBase^15^ and FlyBase^16^ respectively. All genome searches against AGAP009762 were conducted using the National Center for Biotechnology Information’s (NCBI) Basic Local Alignment Search Tool (BLAST). EST data, mRNA sequencing data, and SNAP predictions were assessed in VectorBase^15^,^17^,^18^ and sequence alignments conducted with EBI *ClustalW*.^19^,^20^ Cladograms were created using *ClustalX*^19^ and Dendroscope;^21^ HMM logos produced using WebLogo 3;^22^,^23^ phosphorylation sites predicted with NetPhos 2.0;^24^ and transmembrane domains with TMHMM 2.0.^25^ All gene annotations, primer design, and sequence data processing were conducted using the Geneious^26^ software.

### 5′ and 3′ Random amplification of cDNA ends (RACE) PCR

The Ambion FirstChoice RLM-RACE Kit was used to obtain 5′ and 3′ ds-cDNA regions from AgNimB2 and AgEater; proteins identified by bioinformatics analysis. Primers used were: 5′-RACE-InnerPrimer-AgNimB2: TTTGCGCCACGCGGTCTTTA, 5′-RACE-OuterPrimer-AgNimB2: TGGCTCGGCCATCTCCACAAAA, 3′-RACE-OuterPrimer-AgNimB2: AACGTGCTCCTGTAAGCCTGGGTA, 3′-RACE-InnerPrimer-AgNimB2: GGGGAATGCACTGGACCGAATGTT, 5′-RACE-InnerPrimer-AgEater: ACCGTCACGCGCTTACCATT, 5′-RACE-OuterPrimer-AgEater: TCGATGGTTCGACAGTCGCTCGTA, 3′-RACE-OuterPrimer-AgEater: ACTGCACGAACCAAGTCACAGCA, 3′-RACE-InnerPrimer-AgEater: TCGGCAAGCTCGGTAATGCAGA. Products were cloned into the PCR Blunt Vector and used to transform DH5-alpha cells (Invitrogen, Life Technologies, UK). Following colony selection, the insert was sequenced.

### Mosquito colony maintenance

The *An. gambiae* N’gousso laboratory strain was the reference strain for RNA interference (RNAi) experiments. The colony was raised at 28°C, 70–75% humidity under a 12-hour light/dark cycle and adults maintained on 10% sucrose solution.

### Silencing of AgNimB2 and AgEater in *An. gambiae*

For production of dsRNA, total RNA was extracted from whole, 2-day-old female *An. gambiae* using the Trizol reagent protocol and first strand cDNA synthesized using Superscript III (Invitrogen). PCR products were amplified for AgNimB2 and AgEater using cDNA as the template and primers designed with T7 binding sites (underlined) using *E-RNAi*;^27^ AgNimB2 F:taatacgactcactatagggCCCCGGGTATGAACGTAAT, AgNimB2 R:taatacgactcactatagggGATCGGACAGGTCGGTACAC; AgEater F:taatacgactcactatagggGGTGGAAGAGATCGCTCG, AgEater R:taatacgactcactatagggTCGACACGGTTCCACACATA. The products were then purified (Qiagen PCR purification kit) and used as a template for dsRNA using the T7 MEGAscript kit (Invitrogen). DsRNA was purified (Qiagen RNeasy purification kit, Qiagen, UK), quantitated using a NanoDrop spectrophotometer, and quality confirmed by agarose gel electrophoresis. The final dsRNA concentration was adjusted to 3 μg/μl.

RNA interference was accomplished by injection of 69 nl of dsRNA through the thorax into the hemocoel of anesthetized female mosquitoes using a Nanoject injector (Drummond Scientific, PA, USA). *LacZ* dsRNA was used as a negative control. Mosquitoes were incubated 4 days to allow recovery and efficient gene knockdown (KD). The KD efficiency and gene specificity were confirmed by quantitative real-time PCR (qRT-PCR) following the Takara SYBR Ex *Taq* protocol, using the primers AgNimB2 F: CTGTGTGGATGTGGAGCGAT, AgNimB2 R: TGTGGTGCACACTTGAGCTTCG; AgEater F: TGCTGGGACGGTTACGGAAAGA, AgEater R: CCGGAGCGACACAATCTGCATT.

### *In vivo* phagocytosis assay

69 nl of a 1 mg/ml suspension of pHrodo-labeled *S. aureus* wood strain and *E. coli* K12 bioparticles (Invitrogen) in sterile PBS was injected intrathoracically into the hemocoel. Phagocytosis was visualized 120 minutes post-injection by observing abdominal segments and quantitated by counting the number of pHrodo-labeled bioparticle foci in the abdomen of individual mosquitoes.

### Infection of An. gambiae with Plasmodium berghei

Female *An. gambiae* were fed on WT-mice infected with GFP-expressing *P. berghei* (GFP-CON)-strain using standard laboratory protocols.^28^,^29^ Ten days post-infection mosquito midguts were dissected and oocyst numbers were counted under fluorescent microscopy. 

### Cell line transfection

Full-length (FL) AgNimB2 and AgEater cDNA were amplified using the primers AgNimB2 F: GTTGGGCTGGCTTTCGTGTGC, AgNimB2 R:GGCTCCCTGACTGCCGCTG, and AgEater F: CGGGAGCTGCTCTGTTGGTGG, AgEater R: GGAGGTTTTACGAATATTTTAGGTGTG respectively, and cloned into pCR Blunt vector (Invitrogen). Using the pCR Blunt vector with insert as templates, ligation independent cloning (LIC) primers with 5′ tags (underlined) F: gacgacgacaagatgCAGGAGGGTGTAAAGACCGCG, R: gaggagaagcccggtttTGATTTAACTCGTCGAAGCG, were used to amplify the full open reading frame (ORF) of AgNimB2, excluding the signal peptide and stop codon. Similarly AgEater FL and secreted (SC; without the predicted C-terminal transmembrane domain) forms were amplified using AgEater FL F: gacgacgacaagatgGCCTGCTCGAAAACGAACGTAAAAACG, AgEater FL R: gaggagaagcccggtttGATGGTTTCAATTTCGAGATTCAA; and AgEater SC F: gacgacgacaagatgGCCTGCTCGAAAACGAACGTAAAAACG, AgEater SC R: gaggagaagcccggtttCTTGTAGTACTCGCCACCGGAAC, respectively.

Subsequently, AgNimB2, AgEater-SC, and AgEater-FL LIC PCR products were cloned into the LIC vector, pIEX10 and used to transform NovaBlue GigaSingles (Novagen, Damstadt, Germany). pIEX10 includes an N-terminal Strep epitope tag for immunological detection, a signal peptide, and a C-terminal 10×His tag. Presence of the insert was confirmed by sequencing. Purified plasmid was used to transfect serum-free adapted *Spodoptera frugiperda* Sf9 cell lines (Invitrogen). LRIM1, LRIM15, intracellular GFP (iGFP), and secreted GFP (sGFP) were used as controls.

### Protein localization assays

Four days post-transfection, culture medium and cell lysate from each transfection were visualized on a non-reducing (NR) SDS-PAGE, followed by chemiluminescent western blot (WB) using anti-Strep tag primary antibody and HRP secondary. Mock-transfected cells were used as a negative control and the secreted protein LRIM1^30^,^31^ served as a positive control. Actin was used as a loading control in the cell lysate and Coomassie brilliant blue (G-250) staining conducted to confirm uniform protein loading between culture medium and cell lysate. Western blots were imaged on a Bio-Rad XRS+ system and band intensities were quantitated using ImageLab software (Bio-Rad, CA, USA). Transfected cells were also visualized by immunofluorescence. All cells (except the negative control) were co-transfected with iGFP to indicate successful cell transfection. Additionally, all cells were nuclear stained with DAPI, and Strep tag containing proteins (AgNimB2 and AgEater) identified by an Alex Fluor 568 secondary antibody. The transmembrane protein LRIM15^31^ was used as a positive control.

### Bacteria and ookinete binding assay

Sf9 cell culture medium (500 μl) was obtained 4 days post-transfection from each cell transfection and incubated with 40 μl *E. coli* or *S. aureus* bioparticles (10 mg/ml in PBS with 1% BSA), or 1 μl *P. berghei* ookinetes (500 ookinetes/μl in PBS with 1% BSA) for 1 hour. Ookinetes were purified using 62.5% Nycodenz.^32^ Mixtures of bacteria/ookinetes and conditioned medium were incubated and then centrifuged for 2 minutes at 500 rcf. The supernatant was removed and the pellet was resuspended in PBS. This step was repeated and after the second PBS wash was removed, the pellet was resuspended in 35 μl 5× protein loading buffer and visualized on a NR SDS-PAGE WB. LRIM1 was used as a positive control and sGFP as a negative control. Successful recombinant protein expression in culture medium was confirmed by NR SDS-PAGE WB. An ookinete surface protein Pbs21,^33^ was used as a loading control for ookinete binding assays, and Coomassie stain (G-250) used for bacterial binding blots.

### Data analysis

Statistical and graphical data analysis were conducted using R Statistical Computing Program.^34^

## Results

### AGAP009762 encodes two members of the Nimrod gene superfamily

Detailed expression analyses have been recently published, examining the complexity of the molecular repertoire of *An. gambiae* immune cellular and humoral components.^35^–^39^ To identify novel components of the mosquito anti-bacterial or bacterial-induced immune responses, we searched for genes both upregulated after bacterial challenges and expressed in hemocytes, the main cellular component of mosquito immunity. The following clusters of published microarray catalogs were therefore selected and compared. (i) A group of 107 genes obtained by pooling together two clusters of genes upregulated after bacterial challenge in 4a3B mosquito cell line using both bacterial elicitor Peptidoglycan (PGN) and Lipopolysaccharide (LPS).^38^ These two clusters are enriched in genes belonging to immune functional classes, thus describing a distinctive signature of a core immune response to microbial challenge. (ii) A second group included 297 candidates belonging to two clusters of genes showing specific or enriched expression in mosquito circulating hemocytes when compared to other tissues.^35^ Comparison of these two groups resulted in a list of 10 candidates with the common features of being enriched both in the main immune mosquito tissue (i.e. circulating hemocytes) and upregulated upon bacterial challenge in a cell-based model. Among these, one of the most interesting candidates was *AGAP009762*, predicted to encode a member of the Nimrod superfamily. This gene, located on the right arm of chromosome 3, was originally reported with a length of 3493 bp in the *An. gambiae* PEST genome reference. Since our initial attempts to amplify the predicted AGAP009762 ORF from cDNA failed, we carried out further bioinformatic analyses of ESTs, mRNA deep sequencing data, and SNAP predictions. Taken together, these analyses indicated that the *AGAP009762* annotation was incorrect and that instead the locus contained two separate genes encoding proteins of 390 and 979 amino acids. Basic Local Alignment Search Tool analysis indicated that the smaller protein is orthologous of *Drosophila* Nimrod B2 while the larger is orthologous of *Drosophila* Eater. Henceforth these two proteins are referred to as AgNimB2 and AgEater, respectively.

*AgNimB2* encodes a 1170 base transcript consisting of four exons, while the *AgEater* encodes a 2937 bp transcript consisting of five exons. Random amplification of cDNA ends PCR was used to experimentally confirm both ends of the *AgNimB2* and *AgEater* transcripts (online Supplementary Material 1 and 2) and each predicted ORF was successfully amplified from cDNA (data not shown). We were unable to obtain the entire 5′ region of AgEater, however, the presence of an N-terminal motif (CCxGY) found in members of Nimrod superfamily^40^ supports the prediction.

**Figure 1:**
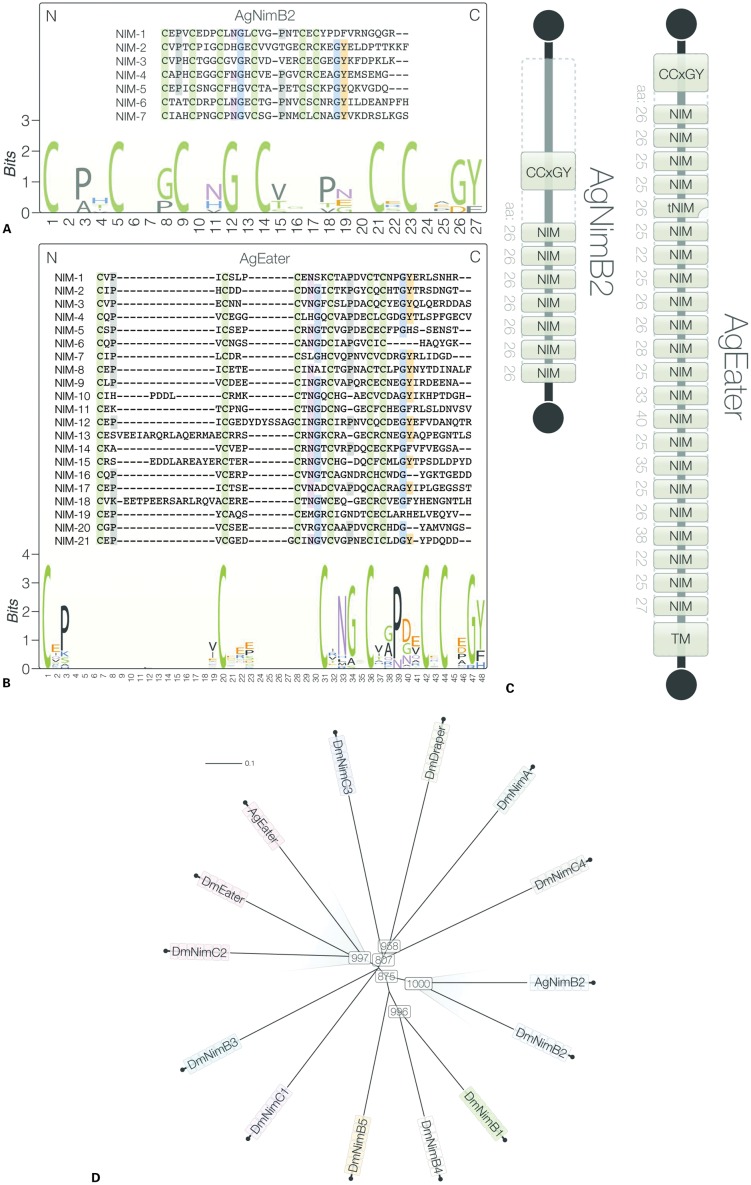
Bioinformatic analysis of AgNimB2 and AgEater (A and B) ClustalW sequence alignment of predicted NIM repeats in AgNimB2 and AgEater with the consensus amino acids highlighted. Both alignments were used to produce a HMM logo representing the conversed amino acids. (C) Diagrammatic representation of the AgNimB2 and AgEater proteins, showing the N- (top) and C-termini (bottom), the transmembrane domain (TM), and NIM repeats. The amino acid length is indicated to the left of each repeat. A truncated NIM repeat in AgEater (tNIM) is represented by a notched rectangle. (D) Unrooted tree generated from the Nimrod superfamily members of *Drosophila melanogaster*, AgNimB2, and AgEater. Orthologs are highlighted in blue (NimB2) and red (Eater). Numbers represent bootstrap values.

Comparable to members of *Drosophila* Nimrod superfamily, both AgNimB2 and AgEater proteins feature repeats of the NIM motif, which are EGF-like repeats consisting six cysteine residues arranged in the conserved CxPxCxxxCxNGxCxxPxxCxCxxGY motif.^13^,^14^,^40^ Using a relaxed consensus, AgNimB2 has seven NIM repeats while AgEater has 21. Alignment of these repeats shows the high level of NIM motif variation within each protein, as also illustrated using a HMM logo (Fig. 1A and B). AgEater additionally has a single truncated repeat containing only two cysteine residues (Fig. 1C). Both proteins contain a signal peptide and a CCxGY motif N-terminal to the first NIM repeat, another characteristic of proteins in the *Drosophila* Nimrod superfamily.^41^ Finally, while AgNimB2 is predicted to be secreted, AgEater contains a predicted transmembrane domain, suggesting it is localized to the cell surface.

PSI-BLAST searches identified another Nimrod superfamily member, AGAP009763. The encoded protein features NIM repeats N-terminally flanked by the characteristic CCxGY motif. This protein is a putative homolog of Drosophila Eater or Nimrod C2 and clusters with AgNimB2 and AgEater by 100% protein coverage. Clustering is also observed within the *Drosophila* Nimrod superfamily^41^ (Fig. 1D).

### AgNimB2 mediates *S. aureus* phagocytosis *in vivo*

Orthologs of AgNimB2 and AgEater in *Drosophila* (DmNimB2 and DmEater) play a role in bacterial phagocytosis.^13^,^14^ DmEater is a membrane-bound phagocytic receptor which directly interacts with Gram-positive and Gram-negative bacteria mediating their phagocytosis by hemocytes.^13^ Much less is known about the binding properties of DmNimB2,^14^ and signaling pathways activated by both genes have not been well characterized to date.^13^,^14^

To investigate the role of AgNimB2 and AgEater in bacterial phagocytosis, we employed a protocol that was successfully used in previous studies.^12^,^42^ After RNAi-mediated silencing of AgNimB2 and AgEater, mosquitoes were injected with *E. coli* and *S. aureus* bioparticles conjugated with pHrodo succinidyl ester, a dye that exhibits stronger fluorescence following the drop of pH in the acidified phagosomes.^43^ Mosquitoes were immobilized 120 minutes after bioparticle injection, mounted onto glass slides, and immediately observed by fluorescence microscopy. Images of mosquito abdomens were captured and analyzed to quantify the fluorescent foci.

**Figure 2:**
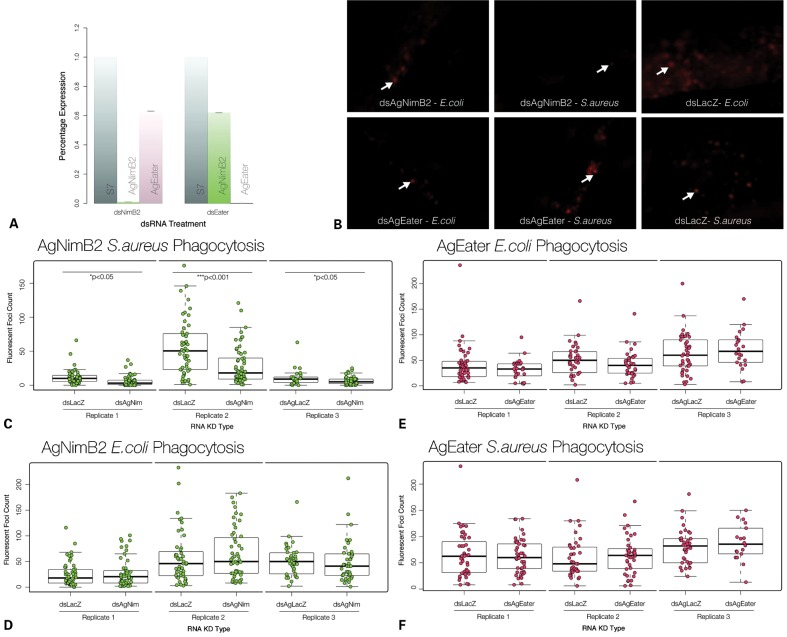
Characterization of the ability of AgNimB2 and AgEater to mediate bacterial phagocytosis. (A) RNA interference (RNAi) gene knockdown (KD) efficiency compared to S7 reference from 20 of each dsRNA injected mosquitoes assayed by quantitative real-time PCR (qRT-PCR). dsAgNim KD efficiency, 99.2±0.02%; dsEater KD efficiency, 99.8±0%. qRT-PCR data analyzed using standard curve method. Error bars represent standard error of the mean for two replicates. (B) Fluorescent images from pHrodo bioparticle injected mosquito abdomens, example of phagocytosis foci are indicated by arrows. (C) pHrodo phagocytosis foci counts from AgNimB2 KD mosquitoes challenged with *Staphylococcus aureus* pHrodo bioparticles, (D) pHrodo phagocytosis foci counts from AgNimB2 KD mosquitoes challenged with *Escherichia coli* pHrodo bioparticles, (E) pHrodo phagocytosis foci counts from AgEater KD mosquitoes challenged with *E. coli* pHrodo bioparticles, (F) pHrodo phagocytosis foci counts from AgEater KD mosquitoes challenged with *S. aureus* pHrodo bioparticles. Each dot represents one mosquito, with three replicates shown for each gene KD. Bar plot indicates median (bold line), inter quartile range (IQR), and 1.5×IQR (dotted line). Medians were compared using a Wilcoxon rank sum test with continuity correction.

Gene silencing efficiently and specifically reduced the expression of *AgNimB2* and *AgEater* without strong cross silencing (Fig. 2A). *AgNimB2* silencing strongly decreased phagocytosis of *S. aureus* bioparticles as shown by the marked reduction of fluorescent foci (Fig. 2B). Compared to ds*LacZ*-treated controls, ds*AgNimB2* treatment resulted in a statistically significant decrease of the median number of foci. Reductions of 70.0, 64.4, and 44.4% were observed in three biological replicates (*w* = 2091.5, *P* = 0.02431; *w* = 1917, *P* = 0.0002274; *w* = 766.5, *P* = 0.03423) (Fig. 2C). This suggests AgNimB2 plays a role in phagocytosis of *S. aureus*. Conversely, phagocytosis of *E. coli* bioparticles was unaffected by AgNimB2 silencing in three biological replicates (*w* = 1382, *P* = 0.8478; *w* = 1088.5, *P* = 0.3445; *w* = 877.5, *P* = 0.3258) (Fig. 2D). Treatment with ds*AgEater* did not affect the level of phagocytosis of either *E. coli* (*w* = 588, *P* = 0.4281; *w* = 540, *P* = 0.1194; *w* = 516, *P* = 0.3523) or *S. aureus* bioparticles (*w* = 1035.5, *P* = 0.8527; *w* = 866, *P* = 0.4017; *w* = 394.5, *P* = 0.3615) compared to ds*LacZ*-treated controls (Fig. 2E and F). These results suggest that either AgEater is not required or plays a redundant role in bacterial phagocytosis. The functional differences between AgNimB2 and AgEater in bacterial phagocytosis provides further experimental evidence that AGAP009762 encodes for two proteins with independent functions.

### AgNimB2 may play a minor role in defense against *P. berghei*

**Figure 3:**
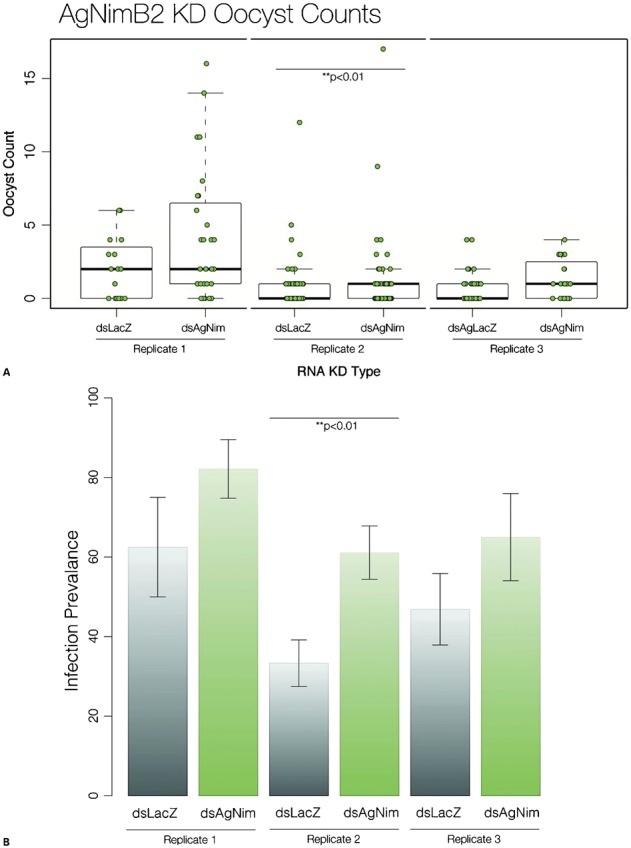
AgNimB2 and AgEaters effect on *Plasmodium berghei* infection. (A) *P. berghei* oocyst counts in AgNimB2 knockdown (KD) and LacZ controls. Each dot represents one mosquito, with three replicates shown for each gene KD. Bar plot indicates median (bold line), inter quartile range (IQR), and 1.5×IQR (dotted line). Medians were compared using a Wilcoxon rank sum test with continuity correction. (B) Mean *Anopheles gambiae* infection prevalence of infected mosquitoes (shown as percentage) for AgNimB2 KD. Means were compared using two sample *t*-test. Errors bars show the standard error of the mean.

Mosquito immune defense against *Plasmodium* has been shown to be tightly linked with the level of basal immunity.^44^ The level of the basal immune system can be modulated by interactions between midgut microbiota before parasite entry.^5^ We have shown above a role of AgNimB2 in *S. aureus* immune defense that may therefore affect immune defense against malaria parasites by modulating basal immune activity. Thus, we evaluated the role of AgNimB2 during the malaria parasite infection in the mosquito. Silencing AgNimB2 led to a significant reduction of the numbers of *P. berghei* oocysts developing in the mosquito midgut in one of three replicates (*w* = −1292.5, *P* = 0.004135) when compared with ds*LacZ*-treated controls (Fig. 3A). In this experiment there was also a significant increase in infection prevalence (61±7% compared to 33±6% of *LacZ* group; *P* = 0.002164, *t* = −3.1358) (Fig. 3B). The other two replicates (1 and 3) showed a similar trend of increased infection levels and prevalence after AgNimB2 silencing, i.e. 20±20% and 18±20%. However, these data were not statistically significant (*w* = 165, *P* = 0.148; *w* = 244.5, *P* = 0.1317) (Fig. 3A and B).

### AgNimB2 is extracellular while AgEater localizes to the cell membrane *in vitro*

Epitope tagged forms of AgNimB2 and AgEater were expressed *in vitro* to investigate their cellular localization. Expression plasmids encoding AgNimB2, AgEater FL, and AgEater SC, which is C-terminally truncated to remove the predicted transmembrane domain (TM) and intracellular portion (AgEater SC) were transfected in *Sf9* insect cells to assess the localization of the corresponding proteins.

**Figure 4:**
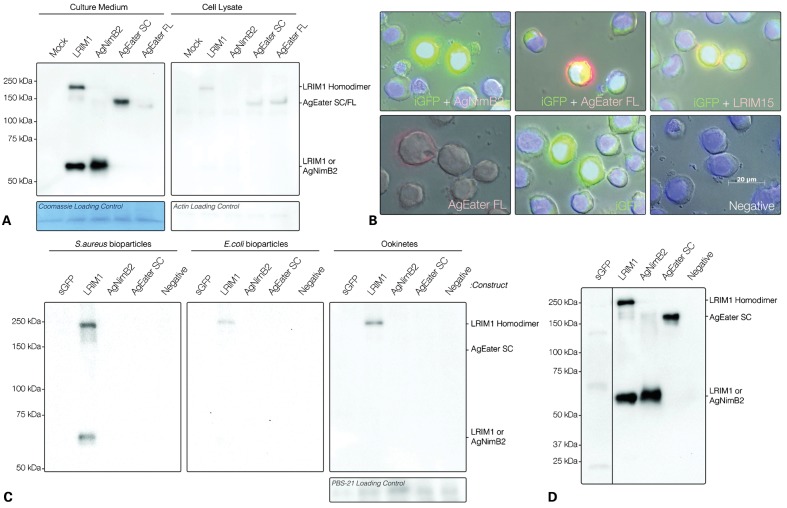
Localization and binding properties of recombinant AGNimB2 and AgEater proteins. (A) A non-reducing SDS-PAGE western blot (WB) of culture medium and cell lysate from transfected Sf9 cell lines, probed using an anti-Strep tag antibody. LRIM1 is expressed in both its dimeric (150 kDa) and monomeric (59.6 kDa) form, rAgNimB2 is monomeric (44 kDa), rAgEater secreted (SC) (97 kDa) and full-length (FL) (109 kDa) are monomeric. As a control, a mock transfection was conducted without DNA. All proteins are secreted into the culture medium, with only LRIM1 and rAgEater present at detectable levels in the cell lysate. Actin was used as a loading control for cell lysate, and Coomassie stains carried out on all samples. (B) Fluorescent images of Sf9 cells transfected with expression plasmids (pIEX10) containing intracellular GFP (iGFP) alone or together with LRIM15, rAgNimB2, rAgEater FL, or mock transfected (Negative). Cells imaged by phase contrast microscopy and fluorescent images overlaid; green: GFP, Red: Strep tag-labeled proteins (LRIM15, rAgNimB2, and rAgEater FL), Blue: DAPI. (D) Non-reducing SDS-PAGE WB probed for Strep tag containing proteins which were selected for binding ability to either *Staphylococcus aureus* bioparticles, *Escherichia coli* bioparticles, or *Plasmodium berghei* ookinetes. Secreted GFP (sGFP) was used as a negative control, LRIM1 as a positive control. Only LRIM1 showed binding to *E. coli*, *S. aureus*, and ookinetes, with only *S. aureus* binding both the LRIM1 monomer and homodimer. PBS-21 was used as a loading control in the ookinete assay. Even loading for all samples was confirmed by Coomassie staining (not shown). (D) Non-reducing SDS-PAGE WB probed for Strep tag containing recombinant protein to confirm their presence in culture medium from transfected sf9 cells (transfected with sGFP, LRIM1, AgNimB2, and AgEater SC) for use in binding assays. All blots are representative of two independent biological replicates.

We used LRIM1, a secreted protein involved in the mosquito complement-like pathway,^30^ as a positive control. Both the monomeric and LRIM1 homodimer are robustly detected in the culture medium of transfected cells (Fig. 4A). It has been shown here that AgNimB2 is also more abundant in the culture medium compared to the cell lysate (the band intensity is over 100-fold higher in the culture medium compared to the cell lysate). Similarly, AgEater SC is abundant in the cell culture medium and compared to the lysate, AgEater SC is approximately eight-fold higher in the culture medium. In contrast, AgEater FL is more abundant in the cell lysate, which supports the *in silico* prediction that it contains a TM. These results provide experimental evidence that AgNimB2 is secreted. The low level of AgNimB2, AgEater SC, and LRIM1 in the cell lysate is consistent with these proteins being in the secretory pathway. AgNimB2, AgEater SC, and AgEater FL migrate at sizes slightly larger than their predicted atomic mass, 58 versus 44, 138 versus 97, and 104 versus 109 kDa, respectively, suggesting that the mature proteins are glycosylated.

Protein localization was also assayed by immunofluorescence of transfected Sf9 cells. AgEater FL localizes to the cell membrane, as evidenced by the red ring around the GFP-expressing cell, whereas AgNimB2 does not in agreement with the western blot data, indicating that AgNimB2 is secreted (Fig. 4B). The transmembrane positive control, LRIM15^31^ was correctly found to be membrane localized.

### AgNimB2 and AgEater do not bind pathogens

DmEater and another member of the *Drosophila* Nimrod superfamily, Nimrod C1, have been shown to bind directly to bacteria.^13^,^14^ We have shown that *in vivo* silencing of AgNimB2 exhibits a decrease in bacterial phagocytosis. To investigate whether AgNimB2 or AgEater bind microbial surfaces, cell conditioned medium containing these proteins were tested in binding assays with *E. coli*, *S. aureus*, or with *Plasmodium* ookinetes.^5^

*E. coli* bioparticles, *S. aureus* bioparticles, or purified ookinetes were incubated with culture medium containing AgNimB2 and AgEater SC. Following incubation, the bioparticles and ookinetes were washed with buffer and then extracted. Analysis of the samples by western blot showed binding only between LRIM1 and *S. aureus*, *E. coli* and ookinetes (Fig. 4C). No binding was detectable for AgNimB2 or AgEater SC and any of the tested surfaces. The presence of similar levels of the recombinant proteins in the starting cell conditioned medium was confirmed (Fig. 4D). These data suggest that AgNimB2 and AgEater do not directly bind bacteria or ookinete surfaces *in vitro*.

## Discussion

Bacterial phagocytosis plays a major role in the cellular immune response of insects.^2^ Its specificity depends on the recognition of microbial patterns by membrane bound or secreted PRRs.^45^ As a result, hemocytes may engulf and kill the pathogen.^2^ This study contributes two novel putative PRRs to the current immune repertoire of *An. gambiae* mosquitoes.

We updated the annotation of predicted *AGAP009762* gene by identifying two orthologs of *Drosophila* phagocytic receptors, *AgNimB2* and *AgEater*. These are the first two members of the Nimrod superfamily described in *An. gambiae*. A common feature of this protein family is the presence of EGF-like NIM repeats C-terminal to a CCxGY motif.^13^,^14^,^40^,^41^ NIM repeats of AgEater show a lower degree of conservation than AgNimB2. The conserved nature of AgNimB2 lends support to the hypothesis that it binds pathogens indirectly, by recognizing/binding conserved host proteins rather than variable pathogen surfaces.

In previous work, dsRNA targeting *AGAP009762* was produced and tested in both *in vivo* and *in vitro* assays.^35^,^42^ It was shown that silencing of AGAP009762/AgEater did not produce any effect on *P. berghei* development in the mosquito.^35^ According to our new prediction, this previously described dsRNA specifically targets AgEater.

We measured *E. coli* and *S. aureus* phagocytosis using pH-dependent fluorescent bioparticles following AgNimB2 and AgEater silencing in female mosquitoes; only AgNimB2 KD showed reduced phagocytosis of *S. aureus* bioparticles. Our studies did not demonstrate a role of AgEater in the phagocytosis of bacteria, suggesting that its function may be redundant or unnecessary in *An. gambiae*, which is in contrast to the function of the *Drosophila* Eater,^13^ which is involved in both *E. coli* and *S. aureus* phagocytosis.^13^,^14^ According to a recent cell-based RNAi screen, no phagocytic activity against *E. coli* was observed targeting AGAP009762/AgEater,^42^ in agreement with the *in vivo* observations presented here. We conclude that while in *Drosophila*, Eater is a well-known hemocyte-specific phagocytic receptor of a broad range of bacteria, including Gram-positives and Gram-negatives, similar function of the *An. gambiae* homolog AGAP009762/AgEater is not observed so far. However simultaneous KD of AgEater and other putative phagocytic receptors would help to investigate AgEater’s role in *E. coli* phagocytosis further.

In contrast, a role of AGAP009762/AgEater in controlling transcriptional activation of the LRIM1 promoter following PGN challenge was recently revealed.^42^ Similarly, a novel activity for DmEater was recently revealed in a screen for novel regulators of JNK following IMD pathway activation upon challenge with PGN in Drosophila cells. It was shown that silencing of Eater caused an increase in activated (phosphorylated) JNK protein, which, in turn, might modulate the expression of Relish-controlled effectors.^46^ It is therefore possible to hypothesize a novel role for Eater orthologs in signal transduction pathways. Pathogen elicitors may be recognized from the cell surface which in turn may block the induced activation of immune pathways to allow the accomplishment of phagocytosis or of some other defense strategies. Finally, in recent publications a further link of DmEater with effector molecules such as AMPs has been described where binding of *E. coli*, *Serratia marcescens* and *Pseudomonas aeruginosa* to Eater is dependent on bacterial membrane disruption by AMPs, indicating cooperation between AMPs and phagocytic receptors during bacterial uptake.^13^,^45^ This suggests a mechanism in which hemocytes/phagocytes are protected from infection of intracellular pathogens by partial or complete bacterial inactivation preceding phagocytosis.

Unlike DmEater,^13^ the role of DmNimB2 during phagocytosis has yet to be investigated. Moreover, members of the Nimrod C1 family were shown to have a greater preference for *S. aureus* phagocytosis compared to *E. coli*.^14^ The phagocytic specificity of AgNimB2 to *S. aureus* likely is a result of binding preference and not a redundant phagocytic pathway for *E. coli*. PAMPs recognized in this phagocytic pathway must be therefore highly specific to Gram-positive bacteria.

**Figure 5:**
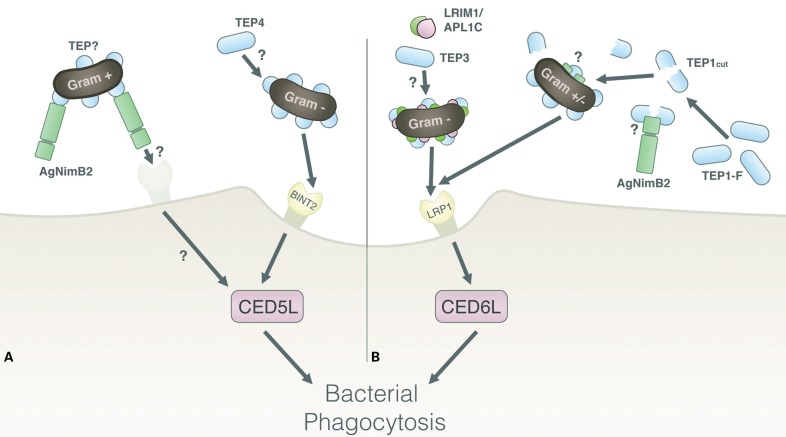
Model of AgNimB2 function in phagocytic pathways of *An. gambiae*. (A) The known BINT2 opsonin-mediated phagocytic pathway, whereby TEP4 acts upstream of BINT2 to mediate phagocytosis of Gram-negative bacteria. One hypothesis for AgNimB2’s action is by binding a TEP-opsonized bacterium and mediating its phagocytosis by then binding a membrane-bound receptor, which will interact with intracellular signaling molecules to mediate phagocytosis, such as CED5L. (B) The known LRP1 opsonin-mediated phagocytic pathway, with TEP3 and LRIM1 acting to mediate Gram-negative bacteria phagocytosis, and TEP1-coated mediating Gram-negative or positive bacterial phagocytosis via LRP1. One hypothesis for AgNimB2’s action is by acting to stabilize and mediate pathogen binding of TEP1 upstream of LRP1 or another pathway. LRP: Lipoteichoic acid recognition protein, TEP: thioester-containing protein.

To date, two phagocytic pathways have been discovered in *An. gambiae* (Fig. 5). Their importance is epitomized by the >50% reduction in phagocytosis of *E. coli* and *S. aureus* following KD of a protein in either pathway.^8^,^12^ One pathway involves the human LDL receptor-related protein 1 (LRP1) ortholog, which features EGF-like repeats,^12^,^47^ and mediates phagocytosis via the intracellular protein CED6L. The other involves a receptor in the beta-integrin family, BINT2, which mediates phagocytosis via the intracellular CED5L.^12^,^48^,^49^ Previous work by Moita and colleagues^12^ highlighted relevant alternatives in the phagocytic pathway of *S. aureus*, since the individual KD of BINT2 or CED5L (proteins in the same signaling pathway) show significantly different phagocytic phenotypes. Indeed BINT2-KD has no effect on *S. aureus* phagocytosis, whereas CED5L KD reduced *S. aureus* phagocytosis by ∼50%. This suggests that another receptor in addition to BINT2 might activate CED5L. It has been shown here that AgNimB2-KD reduces *S. aureus* phagocytosis by ∼50%, thus highlighting the possibility that AgNimB2 could mediate its phagocytic activity via CED5L (Fig. 5A). Co-KD experiments will be needed to investigate this possibility.

Although Eater is strongly implicated in bacterial phagocytosis in *Drosophila*, its role, if any, in this process in *An. gambiae* is unclear. In our silencing experiments, robust KD of AgEater did not have an effect on bacterial phagocytosis suggesting that other than LRP1 and BINT2, no other phagocytic receptors activate CED6L or CED5L. In support of this hypothesis, the fact that the cytoplasmic domain of AgEater lacks NPxY CED6L interaction motifs in the AgEater cytoplasmic domain further confirms this.^50^

We investigated the role of AgNimB2 in protecting the mosquito against *Plasmodium* infection, with the hypothesis that its function in bacterial phagocytosis may play a role in priming the immune response that affects parasites. Results from AgNimB2 KD showed statistical significance in only one of three replicates, suggesting that AgNimB2-mediated bacterial phagocytosis may only have limited involvement in regulating the levels of basal immunity that in turn limits *Plasmodium* infection. Similarly, in *Drosophila* disruption of phagocytosis or silencing of DmEater does not reduce the level of Toll and IMD pathway activation.^13^,^51^

The fact that AgNimB2 is a secreted protein suggests that in order for it to mediate phagocytosis it may directly or indirectly interact with a membrane-bound protein. In *Drosophila*, two proteins containing EGF-like motif, Nimrod C4 and Draper, are secreted and membrane-bound, respectively. Nimrod C4 possibly mediates the recognition and the binding to pathogens, while the activation of CED6 might be mediated by Draper.^2^,^52^ In *Drosophila*, DmNimB2 has been identified to bind at least 10 proteins.^53^–^55^

Neither AgNimB2 nor AgEater directly binds *E. coli*, *S. aureus*, or *P. berghei* ookinetes *in vitro*. In contrast, DmEater has been shown to directly bind both Gram-positive and Gram-negative bacteria.^13^ Moreover, in *Drosophila*, Nimrod C1 does not bind bacteria but mediates phagocytosis, therefore indicating that other hemocyte receptors may play a role in the binding.^14^ The lack of direct binding to ookinetes is not unexpected, as phagocytosis of ookinetes has not been detected *in vivo* as ookinetes rarely come into direct contact with hemocytes. There is a possibility that phagocytosis may play a more important role in sporozoite phagocytosis.^8^

In *An. gambiae*, TEPs are important opsonins of both *P. berghei* and Gram-positive and Gram-negative bacteria.^1^,^31^,^56^ TEP1 KD reduces *E. coli* and *S. aureus* phagocytosis by 60 and 40% respectively.^12^ Thus AgNimB2 may have a role in opsonin-mediated phagocytosis, either in currently identified pathways or in new ones. The leucine-rich repeat protein LRIM1, in complex with APL1C, has been shown to bind TEP1, stabilizing the cut form and this interaction is required for TEP1 localization to the surface of *P. berghei* during infection.^30^,^57^,^58^ In contrast, the phagocytic receptor, LRP1 has been shown to act downstream of complement-like pathway activation, mediating its phagocytic activity by binding TEP1-opsonized bacteria.^12^ Two additional TEP family members, TEP3 and TEP4, were shown to be required for efficient bacterial phagocytosis, presumably through direct interactions with microbial surfaces.^12^ Genetic interactions with LRP1 have been shown for both TEP3 and LRIM1 in phagocytosis of Gram-negative bacteria, whereas the TEP4 has been shown to genetically interact with BINT2.^8^,^12^

We found no evidence that AgNimB2 directly binds bacteria raising the possibility that it mediates its phagocytic activity against *S. aureus* via binding microbial surfaces indirectly by interacting with factors like TEP1 or TEP3. In humans, EGF-like repeat containing LRP1 binds a protein that is a member of the same protein family as TEP’s (alpha_2_-Macroglobulin). Furthermore, human alpha_2_-Macroglobulin does not bind the complement-type repeats found in LRP1, consistent with the possibility that it binds via its EGF-like repeats.^47^ AgNimB2 could balance the lack of LRIM1 activity in the TEP1–LRP–CED6L phagocytic pathway, which is known to stabilize TEP1_cut_ and mediate its pathogen-specific binding in *P. berghei* opsonization^8^,^30^ (Fig. 5). However this does not explain the reason for the *S. aureus* specificity of AgNimB2, unless the lack of *E. coli* activity is due to another redundant pathway for *E. coli* phagocytosis. Additional evidence reveals that TEP1 selectively plays a role in the phagocytosis of *S. aureus* but not of other Gram-positive bacteria.^56^

In conclusion, two new members of Nimrod superfamily AgNimB2 and AgEater have been identified in *An. gambiae* encoding a secreted protein and a membrane-bound protein, respectively. The role of AgNimB2 as a mediator of *S. aureus* phagocytosis, a function likely to occur via an indirect pathway that does not involve direct binding to bacterial surfaces, has been shown. Further work is needed to fully elucidate the molecular pathways by which AgNimB2 mediates its phagocytic effects.
